# Fludarabine melphalan reduced intensity conditioning vs radiation-based myeloablative conditioning in patients undergoing allogeneic transplantation for acute myeloid leukemia with measurable residual disease

**DOI:** 10.1038/s41409-024-02491-0

**Published:** 2024-12-18

**Authors:** Amanda Blackmon, Michelle Afkhami, Dongyun Yang, Sally Mokhtari, Yazeed Samara, Hoda Pourhassan, Brian Ball, Amandeep Salhotra, Vaibhav Agrawal, Karamjeet Sandhu, Amrita Desai, Salman Otoukesh, Shukaib Arslan, Idoroenyi Amanam, Paul Koller, Jose Tinajero, Ahmed Aribi, Ibrahim Aldoss, Pamela Becker, Andy Artz, Haris Ali, Anthony Stein, Eileen Smith, Vinod Pullarkat, Stephen J. Forman, Guido Marcucci, Ryotaro Nakamura, Monzr M. Al Malki

**Affiliations:** 1 : Department of Hematology and Hematopoietic Cell Transplantation, City of Hope National Medical Center, Duarte, CA USA; 2 : Department of Pathology City of Hope National Medical Center, Duarte, CA USA; 3 : Department of Computational and Quantitative Medicine, Beckman Research, Institute of City of Hope, Duarte, CA USA; 4 : Department of Clinical and Translational Project Development, City of Hope National Medical Center, Duarte, CA USA

**Keywords:** Acute myeloid leukaemia, Stem-cell therapies

## Abstract

Patients with AML and measurable residual disease (MRD) undergoing allogeneic hematopoietic cell transplantation (HCT) may benefit from myeloablative conditioning (MAC) when feasible to reduce relapse risk. Fludarabine-Melphalan (FluMel) is a common reduced intensity conditioning (RIC) regimen; however, data in MRD+ patients is sparse. We performed a retrospective review of AML patients who underwent their first HCT (2016–2021) without morphologic disease at City of Hope who had pre-transplant marrow evaluated for MRD using multicolor flow cytometry (MFC) and received radiation-based MAC or FluMel conditioning. We identified 312 patients; 44 with MRD+ disease pre-HCT. The 24-month overall survival (OS), leukemia-free survival (LFS) and cumulative incidence of relapse (CIR) were 47.7%, 40.9%, and 38.6% in MRD+, and 78.0%, 73.9%, and 14.6% in MRD− patients. Radiation-based MAC was given to 136 (43.5%) patients (*n* = 20 with MRD+) and FluMel was given to 174 (55.8%) patients (*n* = 24 with MRD+). In patients with MRD+, there was no statistically significant difference between those who received MAC vs. FluMel in 24-month OS (60% vs. 38%, *p* = 0.21), or CIR (35% vs. 42%, *p* = 0.59), respectively. Our data substantiates the adverse impact of MRD in patients with AML undergoing HCT; FluMel is a reasonable option for MRD+ patients unfit for MAC.

## Introduction

The presence of measurable residual disease (MRD) in acute myeloid leukemia (AML) prior to allogeneic hematopoietic cell transplantation (HCT) has been associated with higher relapse rates and decreased overall survival (OS) using various methods of MRD testing [1–8]. Previous studies have shown conflicting results regarding the differential impact of MRD on transplant outcome according to conditioning intensity in the setting of conventional GvHD prophylaxis platforms, with overall favor of myeloablative conditioning (MAC) when feasible [3, 4, 9, 10]. Hourigan et al. assessed genomic evidence of MRD in AML in a subset of 190 patients enrolled on Blood and Marrow Transplantation Clinical Trials Network (BMT CTN) 0901 with pre-conditioning samples tested for ultra-deep, error corrected sequencing for 13 commonly mutated genes in AML and found a benefit for MAC over RIC for those who tested positive for MRD [8]. A recent study by the Center for International Blood and Marrow Transplant Research (CIBMTR) demonstrated that detectable MRD (by flow cytometry, cytogenetic or molecular techniques) at the time of MAC HCT did not impact outcomes, while detectable MRD preceding RIC HCT was associated with an increased risk of relapse [11]. Likewise, the Acute Leukemia Working Party of the European Society for Blood and Marrow Transplantation (EBMT) showed that RIC/non-MAC was only inferior to MAC regimens for patients in the <50 years old MRD+ group, whereas outcomes of HCT in patients aged ≥50 years old with MRD+ status at HCT were not affected by conditioning intensity; MRD was measured by molecular or immunophenotyping criteria [3].

The impact of MRD in previous studies may be affected by different methods for MRD detection, different conditioning, and possibly GvHD intensity used. The European Leukemia Net update on MRD in AML in 2021 defines MRD positivity in different subsets of disease [12]. In patients without a validated “MRD marker” detectable by quantitative polymerase chain reaction (qPCR) with sensitivity of 10^–6^, the standard MRD testing method is immunophenotyping by multicolor flow cytometry (MFC) with a detection level of 10^−3^. MFC-MRD testing is often sent to the University of Washington, with improved validity due to centralized testing [13].

Fludarabine plus melphalan (FluMel) is commonly used as a reduced toxicity, yet relatively intense regimen. According to a transplant conditioning intensity (TCI) score proposed by the EBMT, which aims to define low, intermediate, and high intensity regimens, FluMel has been assigned as low or intermediate based on melphalan dosing of 110 mg/m^2^ or 140 mg/m^2^ [14]. FluMel performed similarly to myeloablative regimens in another CIBMTR study evaluating conditioning intensity in AML and myelodysplastic syndrome (MDS) with significantly longer relapse-free survival compared to FluBu2+/−ATG, and similar performance to myeloablative Bu4Cy and FluBu4 [15]. Similarly, the PRE-MEASURE study found that patients with MRD by *NPM1/FLT3* next generation sequencing (NGS) had improved survival and lower incidence of relapse with melphalan based conditioning (*n* = 31) as opposed to other reduced intensity regimens (*n* = 21) [16]. Most other studies for MRD + AML include small numbers (less than 20) of patients treated with FluMel for their HCT [3, 8–10, 17, 18] except for a retrospective study by Srour et al., which evaluated AML patients undergoing haploidentical transplant with FluMel and post-transplant cyclophosphamide (PTCy)-based graft-versus-host disease (GVHD) prophylaxis [19]. In this study, outcomes were not influenced by MRD status detected by various methods (*n* = 24 for MRD+) or melphalan dose [19]. In the earlier mentioned BMT CTN 0901 trial, the RIC arm mostly consisted of FluBu2 (classified as lower TCI than FluMel by the EBMT), with a very small portion of FluMel (*n* = 12 with positive MRD) perhaps contributing to worse outcomes with RIC.

In this study, we sought to determine the impact of MRD detected by MFC in patients with AML without morphologic evidence of disease who underwent HCT in our institution considering both the impact of conditioning and GvHD prophylaxis intensity.

## Methods

### Study design and data collection

We identified 312 patients who underwent their first HCT for AML in CR, CR with incomplete count recovery (CRi), or morphologic leukemia-free state (MLFS) at City of Hope between 2016 and 2021. All patients had a pretransplant marrow within 45 days evaluated for MFC-MRD performed at University of Washington [13]. Details of our transplant procedures were described elsewhere [20, 21]. AML risk was according to the ELN 2022 guidelines [22]. HCT-comorbidity index scores were calculated as described by Sorror er al [23]. Patients received matched related, matched unrelated, mismatched unrelated or haploidentical donor transplantation as shown in Table 1.

**Table 1:** Patient and transplant characteristics.

	MRD+ (*N* = 44)	MRD− (*N* = 268)	Total (*N* = 312)	*P* value^a^
Age at HSCT, years				0.065
Median (Range)	61 (22–82)	56 (19–79)	57 (19–82)	
Recipient sex				0.004
Male	33 (75%)	138 (51.5%)	171 (54.8%)	
Female	11 (25%)	130 (48.5%)	141 (45.2%)	
Year of HCT				0.46
2016–2019	23 (52.3%)	156 (58.2%)	179 (57.4%)	
2020–2021	21 (47.7%)	112 (41.8%)	133 (42.6%)	
Female donor to male recipient				0.11
Yes	34 (77.3%)	232 (86.6%)	266 (85.3%)	
No	10 (22.7%)	36 (13.4%)	46 (14.7%)	
Donor age				0.76
Median (Range)	34 (19–66)	34 (12–74)	34 (12–74)	
Disease status at transplant				0.004
1^st^ complete remission	31 (70.5%)	232 (86.6%)	263 (84.3%)	
2^nd^ complete remission	10 (22.7%)	34 (12.7%)	44 (14.1%)	
≥3^rd^ complete remission	3 (6.8%)	2 (0.7%)	5 (1.6%)	
ELN risk				0.052
Favorable	5 (11.4%)	45 (16.8%)	50 (16%)	
Intermediate	13 (29.5%)	117 (43.7%)	130 (41.7%)	
Adverse	26 (59.1%)	106 (39.6%)	132 (42.3%)	
Karnofsky performance status %				0.043
90–100	26 (59.1%)	198 (73.9%)	224 (71.8%)	
≤80	18 (40.9%)	70 (26.1%)	88 (28.2%)	
HCT comorbidity index				0.54
0	7 (15.9%)	61 (22.8%)	68 (21.8%)	
1–2	16 (36.4%)	82 (30.6%)	98 (31.4%)	
≥3	21 (47.7%)	125 (46.6%)	146 (46.8%)	
Donor type				0.26
MRD	11 (25%)	74 (27.6%)	85 (27.2%)	
MUD	19 (43.2%)	135 (50.4%)	154 (49.4%)	
MMUD	7 (15.9%)	19 (7.1%)	26 (8.3%)	
Haplo	7 (15.9%)	40 (14.9%)	47 (15.1%)	
ABO blood group compatibility				0.55
ABO compatible	24 (54.5%)	162 (60.4%)	186 (59.6%)	
Minor mismatch (donor is O)	8 (18.2%)	33 (12.3%)	41 (13.1%)	
Major mismatch (Recipient is O)	8 (18.2%)	38 (14.2%)	46 (14.7%)	
Bidirectional (None are O)	4 (9.1%)	35 (13.1%)	39 (12.5%)	
Donor/Recipient CMV serostatus				0.087
D−/R-	12 (27.3%)	35 (13.1%)	47 (15.1%)	
D−/R+	15 (34.1%)	98 (36.6%)	113 (36.2%)	
D+/R−	4 (9.1%)	23 (8.6%)	27 (8.7%)	
D+/R+	13 (29.5%)	112 (41.8%)	125 (40.1%)	
Conditioning regimen				0.24
Flu/Bu	1 (2.3%)	1 (0.4%)	2 (0.6%)	
FTBI-based	11 (25%)	84 (31.3%)	95 (30.4%)	
TMLI-based	8 (18.2%)	33 (12.3%)	41 (13.1%)	
Melphalan based	24 (54.5%)	150 (56%)	174 (55.8%)	
GVHD prophylaxis				0.76
Tacro/Siro based	28 (63.6%)	177 (66%)	205 (65.7%)	
PTCy based	16 (36.4%)	91 (34%)	107 (34.3%)	

^a^*P* value was based on Wilcoxon two-sample test for continuous variables, chi-square test or Fisher’s exact test for categorical variables.

### Ethics approval and consent to participate

This study was reviewed and approved by the City of Hope Institutional Review Board and was performed in compliance with the Declaration of Helsinki. All methods were performed in accordance with the relevant guidelines and regulations. Due to the retrospective nature of this low-risk non-interventional study, and de-identification of participants, informed consent was deemed not required.

### Treatment plan

Patients received mostly radiation-based MAC (fractionated total body irradiation [FTBI] or total marrow and lymphoid irradiation [TMLI]); or RIC with FluMel. GVHD prophylaxis was either post-transplant cyclophosphamide (PTCy) based or with tacrolimus/sirolimus (tacro/siro) based per institutional standard of practice.

### Response assessment

Response assessments for AML were determined by 2022 Döhner criteria [22].

### MRD assessment

MRD was primarily assessed with multicolor flow cytometry done at the University of Washington as a send out test for uniformity, with any detectable disease considered positive. Exploratory analyses included the presence of additional MRD testing including next generation sequencing (NGS) done at City of Hope, cytogenetics/FISH, and quantitative PCR testing of a validated marker as defined by the ELN MRD guidelines.

### Study endpoints

The primary endpoint was overall survival (OS). Secondary endpoints included leukemia-free survival (LFS), cumulative incidence of relapse (CIR), non-relapse mortality (NRM), as well as the incidence of acute and chronic GVHD. Our primary analysis was to assess the impact of MRD status on transplant outcomes, and secondary objectives included comparison of outcomes of FluMel and radiation-based MAC in patients with MRD, as well as the impact of GvHD prophylaxis intensity.

### Endpoint definitions

Overall survival (OS) was defined as the time from transplant to death due to any cause, or censored on the last known to be alive. Cumulative incidence of relapse (CIR) was defined as time from transplant to morphologic relapse/progression. Non-relapse mortality (NRM) was defined as death from causes not related to disease relapse/progression, and censored at time of last follow-up if patients were alive and remained relapse-/progression-free. Leukemia free survival (LFS) and relapse were defined per CIBMTR criteria and were censored at the time of last follow-up when they remained alive and free of relapse/progression. Grades II–IV and III–IV acute GvHD were defined by the Glucksberg scale, and chronic GvHD was defined as limited or extensive chronic GvHD according to the Seattle criteria.

### Statistical considerations

Descriptive statistics were used to report baseline patient demographic, disease status, MRD status, donor type, conditioning, and GVHD prophylaxis. Two-group Wilcoxon tests, chi-square tests or Fisher’s exact tests, whichever appropriate, were used to compare baseline variables by MRD status. Kaplan–Meier curves and log-rank tests were used to estimate and compare OS and LFS by MRD status in the univariate analysis. Cumulative incidence curves and Gray’s tests were used for CIR and NRM by MRD status. Stepwise Cox regression models and Fine and Gray models were used to build multivariable regression models. The variables associated with an outcome at 0.1 level were included in the final multivariable regression model. All p values were 2 sided at a significant level of 0.05. All analyses were performed using SAS version 9.4 (SAS Institute, Cary, NC). This was a retrospective study with a relatively small sample size. With 44 patients MRD+ and 268 MRD- patients and 107 total events, we had 90% power to detect a difference in OS (if HR= or >2.24) by MRD status using a 2-sided log-rank test.

## Results

### Patients and HCT characteristics

There were 656 consecutive patients who underwent first HCT between 2016 and 2021 for AML in CR, CRi, or MLFS at City of Hope. Of these, 312 had pre-HCT marrow evaluated for MFC-MRD performed at University of Washington and received FluMel or MAC regimen. Patient demographics and transplant characteristics are summarized in Table 1. Briefly, of the 312 patients with available data, 44 (14.1%) had MFC-MRD+ disease on pre-transplant bone marrow assessment. Most patients were in first remission in the entire cohort (84.3%) as well as among MRD+ patients (70.5%). The median age was 61 years old (range: 22–82) in the MRD+ group compared to 56 (19–79) in the MRD- group (*p* = 0.065). Of MRD+ patients, those who received FluMel had a median age of 68 (29–82), compared to 50 (22–73] in the MAC group. Upon stratification of patients for ELN risk, more patients in the adverse risk group had MRD+ prior to transplant (19.7%, *n* = 26/132), compared to intermediate (9.8%, *n* = 13/132) and favorable risk (10%, *n* = 5/50) (*p* = 0.052).

### Transplant outcomes

At the median follow-up of 26.3 months (range:4.4–74) for surviving patients, the estimated 24-month OS, LFS and CIR in those with MFC-MRD+ were 47.7% (95% confidence interval [CI], 32.5–61.5), 40.9% (95% CI, 26.5–54.8), and 38.6% (95% CI, 24.2–52.8); and those with MFC-MRD- were 78.0% (95% CI, 72.5–82.5), 73.9% (95% CI, 68.2–78.7), and 14.6% (95% CI, 10.6–19.1). On multivariate analysis, MFC-MRD+ was independently predictive of worse OS and LFS with hazard ratio (HR) of 2.80 (95% CI, 1.8–4.34; *p* = <0.001) and 2.92 (95 CI, 1.95–4.42; *p* ≤ 0.001), respectively (Table 2). MFC-MRD+ was also associated with higher CIR, HR = 3.13 (95% CI, 1.86–5.26; *p* ≤ 0.001). MFC-MRD+ status was not predictive of non-relapse mortality (NRM) or acute GVHD outcomes. Interestingly, MRD+ was associated with reduced incidence of chronic GVHD (HR = 0.57, 95%CI 0.35–0.93, *p* = 0.028) on univariate analysis.

**Table 2:** Multivariate analysis of transplant outcomes.

			Overall survival		LFS	
		*N*	Adjusted HR (95%CI)^a^	*P*^a^	Adjusted HR (95%CI)^a^	*P*^a^
Disease status	CR1	263	Reference	0.14	Reference	**0.027**
	CR2	49	1.44 (0.89,2.33)		1.63 (1.06,2.52)	
MRD	MRD−	268	Reference	**<0.001**	Reference	**<0.001**
	MRD+	44	2.64 (1.68,4.15)		2.55 (1.66,3.91)	

			Relapse		NRM	
		*N*	Adjusted HR (95%CI)^b^	P^b^	Adjusted HR (95%CI)^b^	P^b^
Disease status	CR1	263	Reference	**0.015**	Reference	0.49
	CR2	49	2.02 (1.15,3.56)		1.26 (0.66,2.41)	
MRD	MRD−	268	Reference	**0.003**	Reference	0.23
	MRD+	44	2.46 (1.36,4.45)		1.54 (0.76,3.11)	

^a^Based on multivariable Cox regression model adjusted for age, KPS and MRD to OS, and KPS, disease status, conditioning, and MRD to LFS.

^b^Based on multivariable Fine and Gray regression model. Disease status, KPS and MRD were adjusted for relapse. Age was adjusted for NRM.

Bold values indicate statistical significance *p* < 0.05.

We examined MRD by other methods including PCR, NGS, or FISH/cytogenetics; 21 of 44 patients with MFC-MRD+ were also MRD+ by an additional method. There were 23 patients with positive MFC-MRD while MRD negative by other methods. There was no difference in LFS or CIR in patients who tested positive by one or more methods (data not shown).

By multivariate analysis, other variables associated with transplant outcomes were; age (≤59 vs ≥60) for OS (HR = 1.64, 95% CI, 1.09–2.49, *P* = −0.031); and KPS (90–100 vs. <90) for OS (HR = 1.63, 95% CI, 1.10–2.42, *p* = 0.013), LFS (HR = 1.66, 95% CI, 1.15–2.39, *p* = 0.006) and CIR (HR = 1.84, 95% CI, 1.13–3.0, *p* = 0.016); conditioning (MAC vs. RIC) for OS (HR = 1.48, 95% CI, 1.00–2.21, *p* = 0.049) but not LFS, CIR, or NRM; remission status (CR1 vs CR2+) for LFS (HR = 1.87, 95% CI, 1.23–2.84, *p* = 0.003), CIR (HR = 2.41, 95%CI, 1.42–4.09, *p* = 0.001), and for grade 2–4 acute GVHD (HR = 1.55, 95% CI, 1.02–2.35, *p* = 0.048) (supplementary Table 2).

### Impact of MRD-MFC in subgroups, conditioning intensity (FluMel and MAC)

**Fig. 1:**
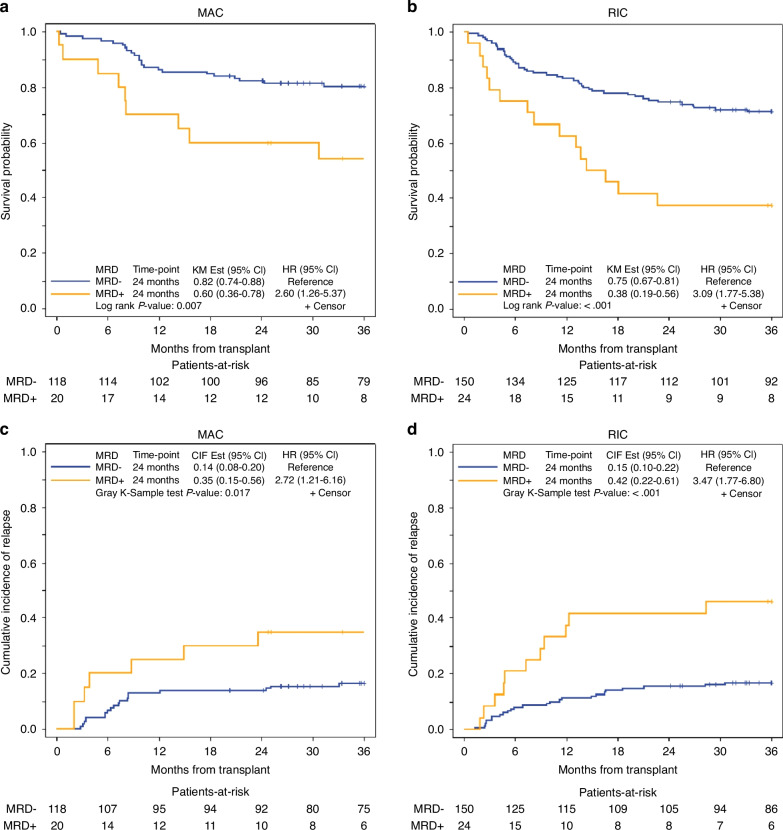
Overall survival and cumulative incidence of relapse by pre-transplant MRD status in patients who received reduced intensity vs. myeloablative conditioning. **a** OS in MRD+ and MRD− patients who received MAC. **b** OS in MRD+ and MRD− patients who received RIC. **c** CIR in MRD+ and MRD− patients who received MAC. **d** CIR in MRD+ and MRD− patients who received RIC.

**Fig. 2:**
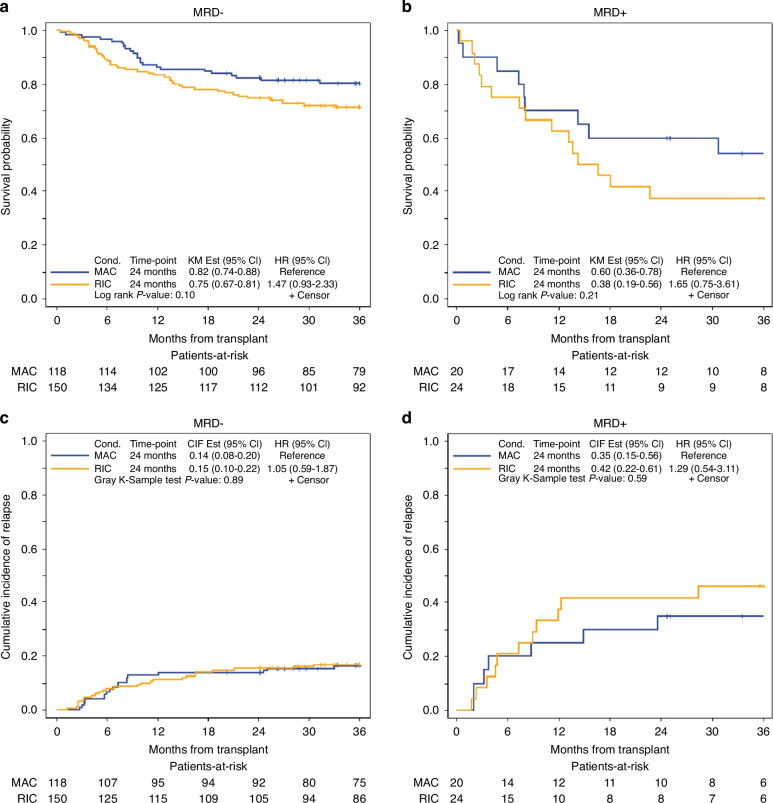
Overall survival and cumulative incidence of relapse by conditioning regimen (RIC vs. MAC) in patients with pre-transplant MRD− vs. MRD+ status. **a** OS of MAC vs. RIC in MRD− patients. **b** OS of MAC vs. RIC in MRD+ patients. **c** CIR of MAC vs. RIC in MRD− patients. **d** CIR of MAC vs. RIC in MRD+ patients.

As shown in supplementary Table 1 conditioning intensity was associated with OS (HR = 1.48, 95% CI, 1.00–2.21, *p* = 0.049) in favor of MAC regardless of MRD status, but there was no statistically significant association with LFS, CIR, or NRM. We examined the impact of MFC-MRD separately in FluMel (*n* = 24 MRD+ and 150 MRD−) and MAC (*n* = 20 MRD+ and 118 MRD−); and showed MRD+ was associated with significantly worse 2-year OS in patients who received both MAC (60% vs. 82%, *p* = 0.007) and FluMel (38% vs. 75%, *p* = <0.001) (Fig. 1a, b, Table 3) with HR of 2.6 and 3.09, respectively. Similarly, higher 2-year CIR was observed in patients with MRD+ who received MAC (35% vs 14%, *p* = 0.017) and FluMel (42% vs. 15%, *p* = <0.001) (Fig. 1c, d, Table 3) with similar HR of 2.72 and 3.47, respectively. Among MFC-MRD+ patients (*n* = 44) there was no significant difference between those who received MAC (*n* = 20) compared with FluMel (*n* = 24) in 24-month OS (60% vs. 38%, *p* = 0.21), or CIR (35% vs. 42%, *p* = 0.59), respectively (Fig. 2).

**Table 3:** Overall survival and cumulative incidence of relapse by conditioning and GVHD prophylaxis.

	Overall survival, 2 year %survival (95% CI)	Cumulative incidence of relapse, 2 year %relapse (95% CI)
Melphalan-based		
All patients (*n* = 174)	69.5% (62.1–75.8)	19.0% (13.5–25.1)
MRD+ (*n* = 24)	38% (19–56)	42% (22–61)
MRD− (*n* = 150)	75% (67–81)	15% (10–22)
Radiation-based MAC		
All patients (*n* = 138)	79.0% (71.2–84.9)	16.7% (11.0–23.4)
MRD+ (*n* = 20)	60% (36–78)	35% (15–56)
MRD− (*n* = 118)	82% (74–88)	14% (8–20)
Tacro/Siro		
All patients (*n* = 205)	73.6% (67.0–79.1)	17.6% (12.7–23.1)
MRD+ (*n* = 28)	61% (40–76)	29% (13–46)
MRD− (*n* = 177)	76% (59–81)	16% (11–22)
PTCy		
All patients (n = 107)	73.8% (64.4–81.1)	18.7% (11.9–26.6)
MRD+ (*n* = 16)	25% (8–47)	56% (27–77)
MRD− (*n* = 91)	82% (73–89)	12% (6–20)

Of patients treated with FluMel with MRD+ disease, 10/24 received melphalan 100 mg/m^2^ and 14 received 140 mg/m^2^. Only 2 patients who received 100 mg/m^2^ are alive at time of analysis; 5 patients experienced relapse and 2 patients died during the peri-transplant period with NRM. No patients received doses of melphalan above 140 mg/m^2^.

### Impact of MRD-MFC in subgroups, GVHD prophylaxis (Tacro/Siro and PTCy)

**Fig. 3:**
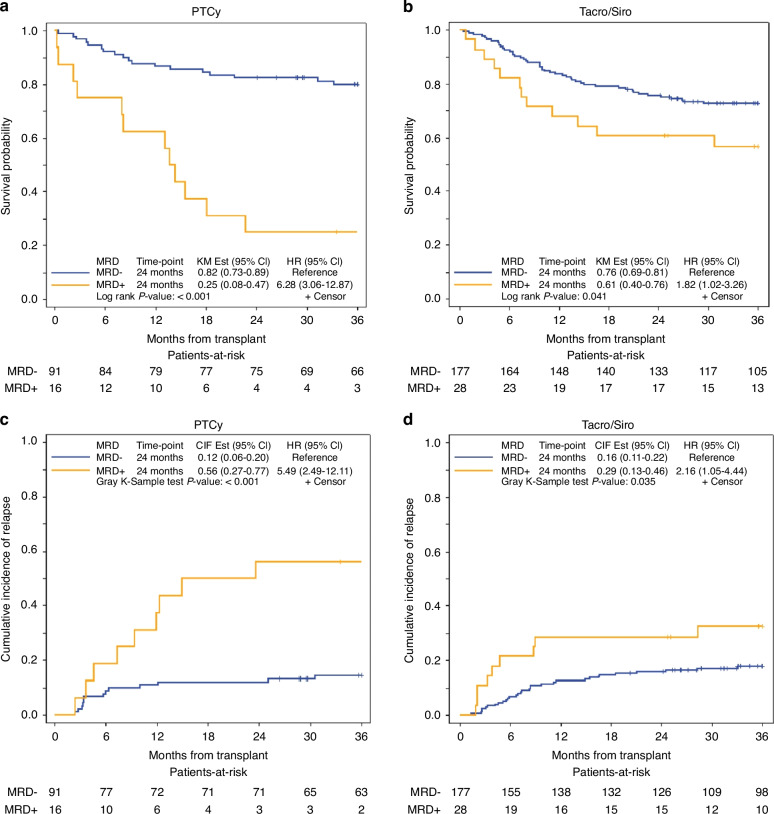
Overall survival and cumulative incidence of relapse by pre-transplant MRD status in patients who received PTCy-based compared to tacrolimus/sirolimus-based GVHD prophylaxis. **a** OS in MRD− and MRD+ patients who received PTCy-based GVHD prophylaxis. **b** OS in MRD− and MRD+ patients who received tacrolimus/sirolimus-based GVHD prophylaxis. **c** CIR in MRD− and MRD+ patients who received PTCy-based GVHD prophylaxis. **d** CIR in MRD− and MRD+ patients who received tacrolimus/sirolimus-based GVHD prophylaxis.

**Fig. 4:**
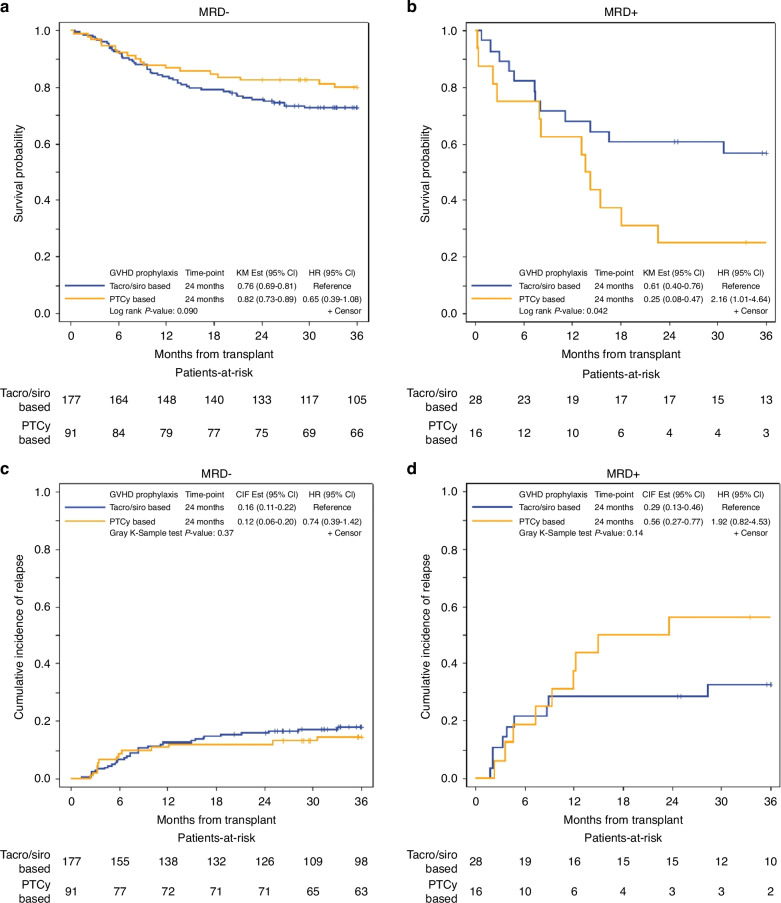
Overall survival and cumulative incidence of relapse by GVHD prophylaxis in patients with pre-transplant MRD− vs. MRD+ status. **a** OS of tacrolimus/sirolimus-based vs. PTCy-based GVHD prophylaxis in MRD− patients. **b** OS of tacrolimus/sirolimus-based vs. PTCy-based GVHD prophylaxis in MRD+ patients. **c** CIR of tacrolimus/sirolimus-based vs. PTCy-based GVHD prophylaxis in MRD− patients. **d** CIR of tacrolimus/sirolimus-based vs. PTCy-based GVHD prophylaxis in MRD+ patients.

The use of tacro/siro or PTCy as GVHD prophylaxis was not associated with significant differences in OS, LFS, or CIR (Supplementary Table 1). We examined the impact of MFC-MRD+ separately in tacro/siro (*n* = 28) and PTCy (*n* = 16). MRD+ was associated with slightly worse OS compared to MRD- in patients who received tacro/siro at 2 years (61% vs. 76.0%, HR 1.82, *p* = 0.041), whereas in those who received PTCy, there was a larger difference (25% vs. 82%, HR 6.28, *p* = <0.001) (Fig. 3a, b, Table 3). Similarly, higher CIR was observed in patients with MRD+ compared to MRD- who received tacro/siro (29% vs 16% at 2-years, *p* = 0.035) and PTCy (56% vs. 12% at 2 years, *p* = <0.001) (Fig. 3c, d, Table 3) with HR or 2.16 and 5.49, respectively. Among MFC-MRD+ patients (*n* = 44), OS was longer in those who received tacro/siro (*n* = 28) compared with PTCy (*n* = 16) with 24-month OS (61% vs. 25%, *p* = 0.042) with HR of 2.16 (Fig. 4a, b); however, there was no significant difference in CIR noting small sample size (29% vs. 56%, *p* = 0.14) (Fig. 4c, d).

### Impact of MRD-MFC in subgroups, ELN risk group

As shown in Table 2, ELN risk stratification was not independently associated with OS, LFS, CIR or NRM. There was a trend towards improved survival in favorable or intermediate risk disease, and similarly a trend towards higher CIR and shorter LFS in those with adverse risk disease. The 2-year CIR in MRD+ vs. MRD- in the intermediate ELN risk group was 36% vs. 12% (95% CI, *p* = 0.004) with HR of 3.69. In the ELN favorable risk group, the 2-year CIR in MRD+ vs MRD- was 80% vs 9% (95% CI, *p* < 0.001) with HR of 15.67. In the ELN adverse risk group, there was no statistically significant difference in the 2-year CIR in MRD+ vs. MRD- with 31% vs. 20% (95% CI, *p* = 0.124) experiencing relapse (Supplementary Fig. 2).

## Discussion

Consistent with earlier reports, our data demonstrated that AML patients with positive MRD by MFC had worse OS and LFS with higher CIR than those who tested MRD negative [1–6]. Our data further the understanding of the impact of MRD in RIC HCT as our cohort had one of the highest number of cases who received FluMel. In our study, the impact of MRD status on transplant outcomes was similar between FluMel and MAC, and patients with MFC-MRD+ undergoing FluMel based conditioning had comparable outcomes to patients undergoing radiation-based MAC. This is probably linked to higher intensity of FluMel compared to other RIC used, especially with standard dosing of most patients with 140 mg/m^2^. While the data need to be interpreted with caution due to the inherent heterogeneity and small sample size (MRD+ cases), our findings are consistent with a report from the CIBMTR that showed comparable relapse-free survival among AML/MDS patients (*N* = 1258) who underwent HCT with FluMel regimen compared to MAC regimens, indicating its efficacy in disease control in myeloid malignancies [15]. There was no MRD data in the CIBMTR study, and our current study data could be considered complementary in this setting.

Our study evaluated MRD by MFC, which is not as sensitive as deeper qPCR based assays standardly used core binding factor leukemia, NPM1-mutated AML, and acute promyelocytic leukemia. The optimal molecular MRD assays in various subsets of disease are still under development and will be further elucidated in the ongoing multicenter MEASURE study, which is prospectively evaluating molecular MRD in patients with AML undergoing transplant [16, 24]. In our study, all patients with MRD+ disease had MFC-MRD positivity detectable at any level – this was at least 0.01% in all patients and while we recognize the sensitivity of the assay is 0.1% it is postulated that the positive predictive value of the test in a centralized setting may be preserved. We evaluated whether test positivity by a second method (qPCR, FISH/cytogenetics, or NGS) influenced HCT outcomes with no impact on relapse or LFS. This is potentially due to the relatively low sensitivity of MFC-MRD compared to other methods at 10^–3^ with molecular methods reaching 10^−6^. Recent molecular MRD data from the SWOG-S0106 study strongly outperformed MFC-MRD in prediction of relapse and survival [25]. As the assays for MRD become more sensitive, the differential impact of conditioning intensity to clear the residual burden of MRD will be of interest, as perhaps a less intense conditioning regimen may be capable of clearing lower-level molecular MRD, and may not clear the relatively higher disease burden of MFC-MRD.

The incorporation of both baseline disease risk (by ELN) and MRD status prior to transplant will be of importance in granular risk-stratification which can potentially inform management decisions including conditioning intensity. In our study, the majority of patients with MRD positive disease had adverse risk AML by ELN criteria (26/44). Previous reports suggest MFC-MRD may be most prognostic in the intermediate risk ELN subgroup, with less impact seen in adverse risk disease; however, some series found MRD was associated with higher relapse incidence across all ELN subgroups [5, 26–28].

The impact of GVHD prophylaxis on relapse risk in AML patients with MRD+ has not been well understood. We attempted to explore potential associations, and while patients who received tacro/siro had longer OS, these data need to be interpreted with caution due to the small number of patients. However, it is plausible that presence of chronic GVHD may possibly be associated with higher GVL in the setting of MRD+ disease, and subsequently better long term control of disease [29]. This suggestion may be more valid when combined with lower intensity conditioning, i.e. FluMel in our study. The effect of GvHD prophylaxis intensity of relapse of those high- risk patients receiving RIC is being should be further studied in a larger and prospective manner (i.e. CTN1703) [30].

We focused on pre-transplant MRD as a one-time static value; however, others have described dynamic/longitudinal monitoring of MRD in addition to chimerism testing to guide pre-emptive management of patients at high risk for relapse. Paras et al., evaluated 810 patients for pre and post-transplantation MRD status who were transplanted either with MAC or non-myeloablative conditioning, and found that peri-HCT dynamics was able to improve risk assessment [31]. Several studies have also looked at post-HCT mixed chimerism as a predictor of relapse and Craddock et al., found that mixed chimerism in patients post-transplant may be a further predictor for relapse and survival in patients with MRD+ status prior to transplant [18]. To follow, Loke et al., reported results from the UK NCRI Figaro Trial showing full T cell chimerism at 3 months post-transplant alleviates the risk of MRD positive disease [32].

Our study has several limitations, mostly attributed to the retrospective nature and the small sample size of patients with MFC-MRD positive disease. This study included patients who received stem cells from multiple donor sources and different GVHD prophylaxis leading to numerous variables possibly confounding the analysis results. Our study included patients without morphologic evidence of leukemia, including those with MLFS where the significance of MRD testing is not well defined. Our study also had a relatively low MRD+ rate at 14.1% with other studies in the literature approach 20% MRD+ prior to transplant [11]. This may be due to technical challenges inherent to MFC testing such as lack of the baseline samples for leukemia-associated immunophenotypes in some or low cellularity/quality of the samples transferred to the central laboratory. It is also possible that the induction/salvage regimens used in our center (or centers that referred patients to City of Hope) were somehow more effective – with increased use of venetoclax based regimens, or with clinical trials [33]. Another limitation is that our study did not assess chimerism post HCT, which may also be predictive of patients at higher risk for relapse.

Well-designed clinical trials are needed to address the management of patients with MRD+ in transplant eligible patients. Potential strategies include additional pre-transplant therapy (post-morphologic remission therapy) to eradicate MRD, escalating conditioning intensity potentially in certain disease subsets or novel targeted conditioning approaches, graft manipulation and preemptive cellular therapy, and post-transplant pharmacologic maintenance therapy when feasible.

In summary, our data substantiates the adverse impact of MRD+ disease in AML, which was similarly observed in patients who received MAC and FluMel-based RIC. The data also supports further efforts on MRD-based risk stratification using molecular-based highly sensitive assays towards additional pre-HCT therapy and/or novel conditioning/post-HCT maintenance approaches.

## Supplementary information

Supplemental Data

## Data Availability

The datasets generated during and/or analyzed during the current study are available from the corresponding author on reasonable request.
