# Supplementary material for: Fludarabine melphalan reduced intensity conditioning vs radiation-based myeloablative conditioning in patients undergoing allogeneic transplantation for acute myeloid leukemia with measurable residual disease
Source: Bone Marrow Transplant. 2024 Dec 18;60(2):165–74. doi: 10.1038/s41409-024-02491-0 (PMC11810767; doi:10.1038/s41409-024-02491-0)
Supplement: Supplementary file 1 — Supplemental Data [file 41409_2024_2491_MOESM1_ESM.docx]

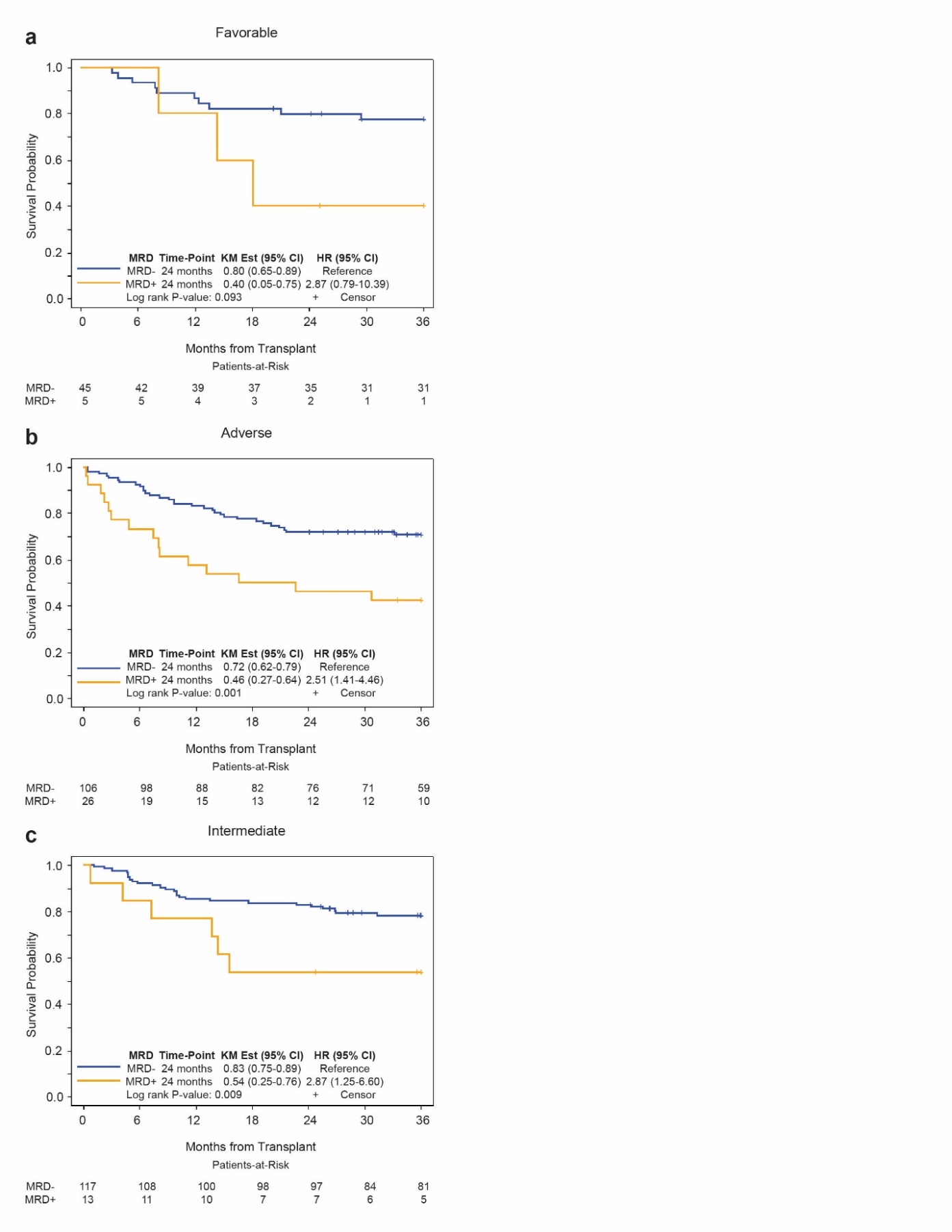
**Supplemental Figure 1.**


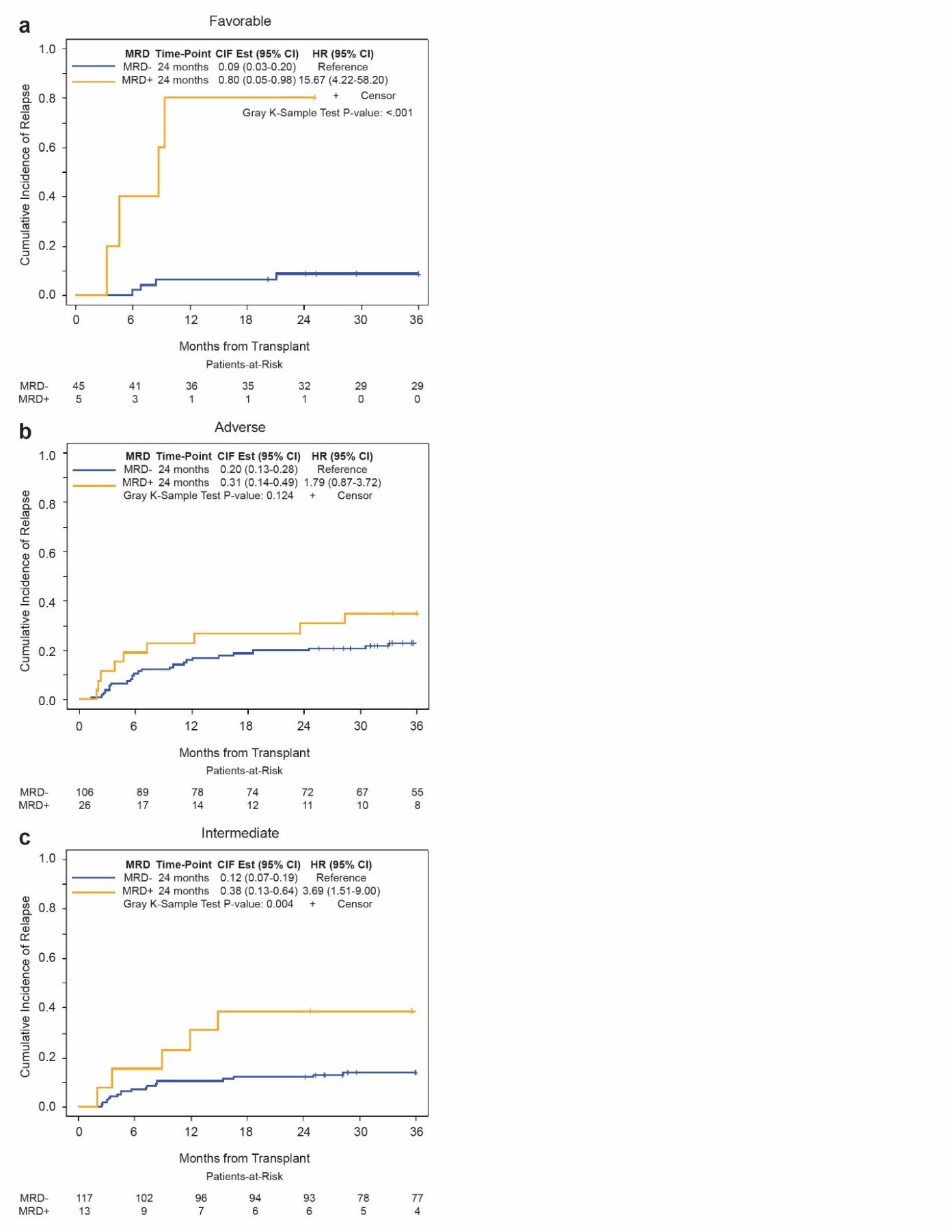
**Supplemental Figure 2.**

**Supplemental Table 1.** Multivariate Analysis of Transplant Outcomes

|  | | | ***Overall Survival*** | | | | | ***LFS*** | | | | |
| --- | --- | --- | --- | --- | --- | --- | --- | --- | --- | --- | --- | --- |
|  |  | *N* | *2 Yr (95%CI)** | *HR (95%CI)** | *P** | *Adjusted HR (95%CI)†* | *P†* | *2 Yr (95%CI)** | *HR (95%CI)** | *P** | *Adjusted HR (95%CI)†* | *P†* |
| **Age at HSCT, years** | ≤59 | 172 | 0.785(0.715,0.839) | Reference | **0.031** | Reference | 0.074 | 0.727(0.653,0.787) | Reference | 0.21 | Reference | 0.76 |
|  | 60-69 | 102 | 0.696(0.597,0.775) | 1.64(1.09,2.49) |  | 1.62(1.06,2.45) |  | 0.667(0.566,0.749) | 1.30(0.88,1.92) |  | 1.16(0.72,1.89) |  |
|  | ≥70 | 38 | 0.632(0.459,0.763) | 1.69(0.97,2.95) |  | 1.38(0.78,2.41) |  | 0.605(0.433,0.740) | 1.49(0.89,2.49) |  | 0.98(0.54,1.80) |  |
| **Sex** | M | 171 | 0.743(0.670,0.802) | Reference | 0.66 | Reference | 0.58 | 0.696(0.621,0.759) | Reference | 0.64 | Reference | 0.42 |
|  | F | 141 | 0.730(0.649,0.796) | 0.92(0.63,1.35) |  | 1.12(0.75,1.66) |  | 0.688(0.604,0.757) | 0.92(0.64,1.31) |  | 1.16(0.80,1.69) |  |
| **Disease Status** | CR1 | 263 | 0.749(0.692,0.797) | Reference | 0.066 | Reference | 0.14 | 0.726(0.668,0.776) | Reference | **0.003** | Reference | **0.027** |
|  | CR2+ | 49 | 0.673(0.523,0.786) | 1.54(0.97,2.44) |  | 1.44(0.89,2.33) |  | 0.510(0.364,0.639) | 1.87(1.23,2.84) |  | 1.63(1.06,2.52) |  |
| **ELN** | Favorable | 50 | 0.759(0.615,0.856) | Reference | 0.067 | Reference | 0.32 | 0.679(0.530,0.789) | Reference | 0.12 | Reference | 0.41 |
|  | Intermediate | 130 | 0.800(0.720,0.859) | 1.01(0.55,1.83) |  | 1.01(0.56,1.85) |  | 0.762(0.679,0.826) | 0.99(0.57,1.71) |  | 1.07(0.61,1.87) |  |
|  | Adverse | 132 | 0.667(0.579,0.740) | 1.57(0.88,2.78) |  | 1.36(0.76,2.44) |  | 0.629(0.540,0.705) | 1.44(0.85,2.44) |  | 1.35(0.78,2.33) |  |
| **KPS** | 90-100 | 224 | 0.781(0.721,0.830) | Reference | **0.013** | Reference | 0.099 | 0.737(0.674,0.789) | Reference | **0.006** | Reference | 0.091 |
|  | ≤80 | 88 | 0.625(0.515,0.717) | 1.63(1.10,2.42) |  | 1.40(0.94,2.09) |  | 0.580(0.470,0.675) | 1.66(1.15,2.39) |  | 1.39(0.95,2.03) |  |
| **HCTCI** | 0 | 68 | 0.794(0.677,0.873) | Reference | 0.12 | Reference | 0.19 | 0.750(0.629,0.836) | Reference | 0.12 | Reference | 0.44 |
|  | 1-2 | 98 | 0.796(0.701,0.863) | 1.26(0.71,2.24) |  | 1.08(0.60,1.93) |  | 0.755(0.657,0.828) | 1.39(0.81,2.38) |  | 1.21(0.70,2.08) |  |
|  | ≥3 | 146 | 0.671(0.589,0.741) | 1.67(0.98,2.85) |  | 1.50(0.88,2.58) |  | 0.623(0.539,0.696) | 1.67(1.01,2.77) |  | 1.39(0.83,2.32) |  |
| **Conditioning** | MAC | 138 | 0.790(0.712,0.849) | Reference | **0.049** | Reference | 0.48 | 0.746(0.665,0.811) | Reference | 0.086 | Reference | 0.072 |
|  | RIC/NMA | 174 | 0.695(0.621,0.758) | 1.48(1.00,2.21) |  | 1.21(0.72,2.02) |  | 0.649(0.574,0.715) | 1.37(0.95,1.98) |  | 1.41(0.97,2.05) |  |
| **Donor** | Matched | 239 | 0.732(0.671,0.784) | Reference | 0.79 | Reference | 0.96 | 0.690(0.627,0.745) | Reference | 0.68 | Reference | 0.98 |
|  | Not | 73 | 0.753(0.638,0.837) | 0.94(0.60,1.48) |  | 0.99(0.62,1.57) |  | 0.699(0.579,0.790) | 0.92(0.60,1.40) |  | 0.99(0.64,1.54) |  |
| **F to M HCT** | No | 266 | 0.748(0.691,0.796) | Reference | 0.22 | Reference | 0.43 | 0.699(0.640,0.751) | Reference | 0.18 | Reference | 0.77 |
|  | Yes | 46 | 0.674(0.519,0.789) | 1.35(0.83,2.20) |  | 1.22(0.75,2.01) |  | 0.652(0.496,0.770) | 1.36(0.86,2.14) |  | 1.07(0.67,1.73) |  |
| **ABO** | Compatible | 186 | 0.769(0.701,0.823) | Reference | 0.23 | Reference | 0.15 | 0.720(0.650,0.779) | Reference | 0.11 | Reference | 0.18 |
|  | Minor | 41 | 0.683(0.517,0.802) | 1.45(0.84,2.49) |  | 1.44(0.84,2.49) |  | 0.610(0.444,0.740) | 1.60(0.98,2.62) |  | 1.52(0.92,2.50) |  |
|  | Major | 46 | 0.761(0.610,0.860) | 1.04(0.59,1.83) |  | 1.01(0.57,1.79) |  | 0.761(0.610,0.860) | 1.01(0.59,1.72) |  | 0.96(0.56,1.64) |  |
|  | Bidirectional | 39 | 0.615(0.445,0.747) | 1.63(0.95,2.80) |  | 1.78(1.03,3.07) |  | 0.564(0.396,0.702) | 1.60(0.96,2.66) |  | 1.55(0.93,2.59) |  |
| **CMV** | D-/R- | 47 | 0.766(0.617,0.863) | Reference | 0.69 | Reference | 0.49 | 0.681(0.527,0.794) | Reference | 0.52 | Reference | 0.87 |
|  | D-/R+ | 113 | 0.726(0.633,0.798) | 1.10(0.61,1.98) |  | 1.48(0.80,2.75) |  | 0.681(0.587,0.759) | 0.88(0.53,1.48) |  | 1.02(0.60,1.72) |  |
|  | D+/R- | 27 | 0.667(0.457,0.811) | 1.28(0.59,2.80) |  | 1.76(0.79,3.91) |  | 0.667(0.457,0.811) | 0.89(0.43,1.85) |  | 1.01(0.48,2.11) |  |
|  | D+/R+ | 125 | 0.752(0.666,0.819) | 0.90(0.49,1.63) |  | 1.29(0.69,2.41) |  | 0.712(0.624,0.783) | 0.70(0.41,1.18) |  | 0.86(0.50,1.48) |  |
| **GVHD prophylaxis** | T/S | 205 | 0.736(0.670,0.791) | Reference | 0.53 | Reference | 0.94 | 0.693(0.624,0.751) | Reference | 0.54 | Reference | 0.99 |
|  | PTCy | 107 | 0.738(0.644,0.811) | 0.88(0.58,1.32) |  | 0.98(0.65,1.49) |  | 0.692(0.595,0.770) | 0.89(0.60,1.30) |  | 1.00(0.68,1.48) |  |
| **Year of HCT** | 2016-2019 | 179 | 0.760(0.690,0.816) | Reference | 0.30 | Reference | 0.25 | 0.721(0.649,0.780) | Reference | 0.18 | Reference | 0.22 |
|  | 2020-2021 | 133 | 0.707(0.621,0.776) | 1.22(0.81,1.82) |  | 1.27(0.84,1.91) |  | 0.654(0.567,0.728) | 1.27(0.88,1.84) |  | 1.27(0.87,1.84) |  |
| **MRD** | MRD- | 268 | 0.780(0.725,0.825) | Reference | **<0.001** | Reference | **<0.001** | 0.739(0.682,0.787) | Reference | **<0.001** | Reference | **<0.001** |
|  | MRD+ | 44 | 0.477(0.325,0.615) | 2.80(1.80,4.34) |  | 2.64(1.68,4.15) |  | 0.409(0.265,0.548) | 2.93(1.95,4.42) |  | 2.55(1.66,3.91) |  |

* Based on univariate analysis and log-rank test

† Based on multivariable Cox regression model adjusted for age, KPS and MRD to OS, and KPS, disease status, conditioning, and MRD to LFS.

|  | | | ***Relapse*** | | | | | ***NRM*** | | | | |
| --- | --- | --- | --- | --- | --- | --- | --- | --- | --- | --- | --- | --- |
|  |  | *N* | *2 Yr (95%CI)** | *HR (95%CI)** | *P** | *Adjusted HR (95%CI)†* | *P†* | *100 day (95%CI)** | *HR (95%CI)** | *P** | *Adjusted HR (95%CI)†* | *P†* |
| **Age at HSCT, years** | ≤59 | 172 | 0.175(0.122,0.235) | Reference | 0.69 | Reference | 0.89 | 0.023(0.008,0.055) | Reference | 0.082 | Reference | 0.078 |
|  | 60-69 | 102 | 0.167(0.102,0.246) | 0.86(0.49,1.51) |  | 0.87(0.48,1.57) |  | 0.069(0.030,0.129) | 1.86(1.05,3.30) |  | 1.86(1.05,3.30) |  |
|  | ≥70 | 38 | 0.237(0.116,0.382) | 1.20(0.61,2.38) |  | 0.91(0.45,1.85) |  | 0.053(0.009,0.157) | 1.77(0.83,3.76) |  | 1.77(0.83,3.76) |  |
| **Sex** | M | 171 | 0.158(0.108,0.217) | Reference | 0.45 | Reference | 0.056 | 0.058(0.030,0.101) | Reference | 0.18 | Reference | 0.24 |
|  | F | 141 | 0.206(0.143,0.276) | 1.20(0.75,1.94) |  | 1.63(0.99,2.69) |  | 0.021(0.006,0.056) | 0.69(0.41,1.18) |  | 0.73(0.43,1.24) |  |
| **Disease Status** | CR1 | 263 | 0.144(0.105,0.190) | Reference | **0.001** | Reference | **0.015** | 0.046(0.025,0.076) | Reference | 0.71 | Reference | 0.49 |
|  | CR2+ | 49 | 0.367(0.234,0.502) | 2.41(1.42,4.09) |  | 2.02(1.15,3.56) |  | 0.020(0.002,0.095) | 1.14(0.60,2.17) |  | 1.26(0.66,2.41) |  |
| **ELN** | Favorable | 50 | 0.161(0.074,0.276) | Reference | 0.17 | Reference | 0.38 | 0.020(0.002,0.093) | Reference | 0.81 | Reference | 0.89 |
|  | Intermediate | 130 | 0.146(0.092,0.213) | 1.18(0.53,2.63) |  | 1.24(0.55,2.77) |  | 0.023(0.006,0.061) | 0.83(0.39,1.74) |  | 0.84(0.40,1.77) |  |
|  | Adverse | 132 | 0.220(0.153,0.294) | 1.77(0.82,3.83) |  | 1.66(0.74,3.73) |  | 0.068(0.033,0.120) | 1.00(0.48,2.06) |  | 0.94(0.45,1.95) |  |
| **KPS** | 90-100 | 224 | 0.152(0.108,0.202) | Reference | **0.016** | Reference | 0.073 | 0.036(0.017,0.066) | Reference | 0.49 | Reference | 0.58 |
|  | ≤80 | 88 | 0.250(0.165,0.344) | 1.84(1.13,3.00) |  | 1.61(0.96,2.71) |  | 0.057(0.021,0.119) | 1.26(0.72,2.21) |  | 1.17(0.67,2.06) |  |
| **HCTCI** | 0 | 68 | 0.162(0.086,0.259) | Reference | 0.44 | Reference | 0.75 | 0.015(0.001,0.071) | Reference | 0.11 | Reference | 0.11 |
|  | 1-2 | 98 | 0.173(0.106,0.255) | 1.58(0.77,3.22) |  | 1.27(0.60,2.71) |  | 0.010(0.001,0.050) | 1.14(0.51,2.56) |  | 1.11(0.49,2.50) |  |
|  | ≥3 | 146 | 0.192(0.132,0.260) | 1.32(0.66,2.65) |  | 1.06(0.53,2.14) |  | 0.075(0.040,0.126) | 1.87(0.90,3.88) |  | 1.85(0.89,3.82) |  |
| **Conditioning** | MAC | 138 | 0.167(0.110,0.234) | Reference | 0.72 | Reference | 0.68 | 0.029(0.010,0.068) | Reference | 0.073 | Reference | 0.70 |
|  | RIC/NMA | 174 | 0.190(0.135,0.251) | 1.10(0.67,1.78) |  | 1.11(0.66,1.87) |  | 0.052(0.025,0.092) | 1.69(0.97,2.94) |  | 1.19(0.49,2.86) |  |
| **Donor** | Matched | 239 | 0.176(0.130,0.227) | Reference | 0.91 | Reference | 0.91 | 0.042(0.021,0.073) | Reference | 0.51 | Reference | 0.66 |
|  | Not | 73 | 0.192(0.111,0.290) | 1.03(0.59,1.80) |  | 0.97(0.56,1.66) |  | 0.041(0.011,0.105) | 0.80(0.41,1.54) |  | 0.86(0.44,1.68) |  |
| **F to M HCT** | No | 266 | 0.173(0.130,0.221) | Reference | 0.23 | Reference | 0.87 | 0.045(0.025,0.075) | Reference | 0.67 | Reference | 0.61 |
|  | Yes | 46 | 0.217(0.111,0.346) | 1.46(0.80,2.64) |  | 1.06(0.54,2.08) |  | 0.022(0.002,0.101) | 1.20(0.61,2.36) |  | 1.20(0.60,2.37) |  |
| **ABO** | Compatible | 186 | 0.167(0.117,0.224) | Reference | 0.76 | Reference | 0.88 | 0.043(0.020,0.079) | Reference | 0.26 | Reference | 0.19 |
|  | Minor | 41 | 0.244(0.125,0.384) | 1.40(0.71,2.76) |  | 1.27(0.64,2.52) |  | 0.024(0.002,0.112) | 1.66(0.83,3.33) |  | 1.67(0.83,3.36) |  |
|  | Major | 46 | 0.152(0.066,0.271) | 0.95(0.46,1.96) |  | 0.95(0.48,1.88) |  | 0.043(0.008,0.132) | 1.13(0.52,2.44) |  | 1.14(0.52,2.51) |  |
|  | Bidirectional | 39 | 0.205(0.095,0.344) | 1.19(0.57,2.49) |  | 1.16(0.53,2.54) |  | 0.051(0.009,0.153) | 1.87(0.91,3.85) |  | 2.05(0.99,4.25) |  |
| **CMV** | D-/R- | 47 | 0.191(0.094,0.316) | Reference | 0.42 | Reference | 0.77 | 0.064(0.016,0.159) | Reference | 0.37 | Reference | 0.39 |
|  | D-/R+ | 113 | 0.150(0.092,0.223) | 0.60(0.31,1.16) |  | 0.71(0.36,1.40) |  | 0.053(0.022,0.106) | 1.52(0.66,3.53) |  | 1.51(0.66,3.48) |  |
|  | D+/R- | 27 | 0.259(0.112,0.435) | 0.90(0.36,2.24) |  | 0.94(0.42,2.10) |  | 0.0 (no events) | 0.96(0.29,3.22) |  | 1.05(0.32,3.46) |  |
|  | D+/R+ | 125 | 0.184(0.122,0.257) | 0.66(0.35,1.25) |  | 0.79(0.41,1.54) |  | 0.032(0.010,0.074) | 0.90(0.37,2.18) |  | 0.92(0.39,2.21) |  |
| **GVHD prophylaxis** | T/S | 205 | 0.176(0.127,0.231) | Reference | 0.97 | Reference | 0.92 | 0.034(0.015,0.066) | Reference | 0.31 | Reference | 0.39 |
|  | PTCy | 107 | 0.187(0.119,0.266) | 1.01(0.61,1.67) |  | 1.02(0.63,1.67) |  | 0.056(0.023,0.111) | 0.73(0.40,1.32) |  | 0.76(0.42,1.41) |  |
| **Year of HCT** | 2016-2019 | 179 | 0.162(0.112,0.220) | Reference | 0.28 | Reference | 0.36 | 0.034(0.014,0.068) | Reference | 0.62 | Reference | 0.86 |
|  | 2020-2021 | 133 | 0.203(0.139,0.275) | 1.28(0.79,2.06) |  | 1.25(0.77,2.03) |  | 0.053(0.023,0.100) | 1.04(0.61,1.78) |  | 1.05(0.61,1.80) |  |
| **MRD** | MRD- | 268 | 0.146(0.106,0.191) | Reference | **<0.001** | Reference | **0.003** | 0.030(0.014,0.056) | Reference | 0.16 | Reference | 0.23 |
|  | MRD+ | 44 | 0.386(0.242,0.528) | 3.13(1.86,5.26) |  | 2.46(1.36,4.45) |  | 0.114(0.041,0.227) | 1.62(0.82,3.18) |  | 1.54(0.76,3.11) |  |

* Based on univariate analysis and Gray’s test

† Based on multivariable Fine and Gray regression model. Disease status, KPS and MRD were adjusted for relapse. Age was adjusted for NRM.

|  | | | ***Grade II-IV aGVHD*** | | | | | ***Grade III-IV aGVHD*** | | | | |
| --- | --- | --- | --- | --- | --- | --- | --- | --- | --- | --- | --- | --- |
|  |  | *N* | *100 days (95%CI)** | *HR (95%CI)** | *P** | *Adjusted HR (95%CI)†* | *P†* | *100 days (95%CI)** | *HR (95%CI)** | *P** | *Adjusted HR (95%CI)†* | *P†* |
| **Age at HSCT, years** | ≤59 | 172 | 0.378(0.305,0.450) | Reference | 0.43 | Reference | 0.58 | 0.110(0.069,0.163) | Reference | 0.91 | Reference | 0.95 |
|  | 60-69 | 102 | 0.307(0.220,0.398) | 0.79(0.53,1.18) |  | 0.82(0.55,1.24) |  | 0.129(0.072,0.202) | 1.10(0.56,2.17) |  | 1.06(0.54,2.11) |  |
|  | ≥70 | 38 | 0.263(0.135,0.410) | 0.69(0.35,1.39) |  | 0.79(0.39,1.60) |  | 0.105(0.033,0.227) | 0.96(0.32,2.90) |  | 0.89(0.29,2.67) |  |
| **Sex** | M | 171 | 0.322(0.253,0.392) | Reference | 0.28 | Reference | 0.52 | 0.123(0.079,0.177) | Reference | 0.70 | Reference | 0.85 |
|  | F | 141 | 0.364(0.285,0.444) | 1.23(0.86,1.76) |  | 1.13(0.78,1.63) |  | 0.107(0.063,0.165) | 0.91(0.49,1.67) |  | 0.94(0.51,1.74) |  |
| **Disease Status** | CR1 | 263 | 0.317(0.261,0.374) | Reference | **0.048** | Reference | 0.052 | 0.118(0.083,0.161) | Reference | 0.69 | Reference | 0.65 |
|  | CR2+ | 49 | 0.469(0.324,0.602) | 1.55(1.02,2.35) |  | 1.49(1.00,2.22) |  | 0.102(0.037,0.206) | 0.81(0.32,2.02) |  | 0.81(0.33,2.01) |  |
| **ELN** | Favorable | 50 | 0.540(0.391,0.668) | Reference | **0.002** | Reference | **<0.001** | 0.120(0.048,0.227) | Reference | 0.93 | Reference | 0.87 |
|  | Intermediate | 130 | 0.310(0.232,0.391) | 0.49(0.31,0.77) |  | 0.49(0.31,0.77) |  | 0.109(0.062,0.169) | 0.85(0.36,1.97) |  | 0.83(0.36,1.94) |  |
|  | Adverse | 132 | 0.295(0.220,0.375) | 0.44(0.27,0.70) |  | 0.44(0.27,0.70) |  | 0.121(0.072,0.183) | 0.94(0.41,2.17) |  | 0.98(0.42,2.28) |  |
| **KPS** | 90-100 | 224 | 0.345(0.283,0.408) | Reference | 0.58 | Reference | 0.67 | 0.108(0.071,0.152) | Reference | 0.82 | Reference | 0.70 |
|  | ≤80 | 88 | 0.330(0.233,0.429) | 0.87(0.57,1.31) |  | 0.91(0.59,1.40) |  | 0.136(0.074,0.217) | 1.09(0.56,2.15) |  | 1.14(0.58,2.23) |  |
| **HCTCI** | 0 | 68 | 0.284(0.181,0.395) | Reference | 0.29 | Reference | 0.40 | 0.075(0.027,0.154) | Reference | 0.31 | Reference | 0.25 |
|  | 1-2 | 98 | 0.296(0.209,0.388) | 0.99(0.59,1.67) |  | 1.05(0.62,1.77) |  | 0.092(0.045,0.159) | 1.11(0.42,2.92) |  | 1.02(0.39,2.65) |  |
|  | ≥3 | 146 | 0.397(0.317,0.476) | 1.30(0.81,2.09) |  | 1.32(0.82,2.12) |  | 0.151(0.098,0.214) | 1.75(0.74,4.18) |  | 1.71(0.73,4.04) |  |
| **Conditioning** | MAC | 138 | 0.384(0.303,0.465) | Reference | 0.21 | Reference | 0.19 | 0.094(0.053,0.150) | Reference | 0.32 | Reference | 0.49 |
|  | RIC/NMA | 174 | 0.306(0.239,0.376) | 0.78(0.54,1.11) |  | 0.78(0.55,1.12) |  | 0.133(0.087,0.188) | 1.35(0.73,2.51) |  | 1.25(0.67,2.35) |  |
| **Donor** | Matched | 239 | 0.361(0.301,0.422) | Reference | 0.13 | Reference | 0.11 | 0.126(0.088,0.172) | Reference | 0.16 | Reference | 0.18 |
|  | Not | 73 | 0.274(0.177,0.380) | 0.69(0.43,1.09) |  | 0.68(0.43,1.08) |  | 0.082(0.033,0.160) | 0.57(0.25,1.30) |  | 0.57(0.25,1.30) |  |
| **F to M HCT** | No | 266 | 0.343(0.287,0.401) | Reference | 0.35 | Reference | 0.39 | 0.106(0.072,0.146) | Reference | 0.26 | Reference | 0.30 |
|  | Yes | 46 | 0.326(0.196,0.463) | 0.80(0.48,1.34) |  | 0.80(0.48,1.33) |  | 0.174(0.081,0.297) | 1.53(0.74,3.16) |  | 1.48(0.71,3.09) |  |
| **ABO** | Compatible | 186 | 0.351(0.283,0.420) | Reference | 0.94 | Reference | 0.87 | 0.124(0.082,0.176) | Reference | 0.84 | Reference | 0.79 |
|  | Minor | 41 | 0.341(0.201,0.487) | 0.94(0.54,1.65) |  | 0.94(0.54,1.62) |  | 0.122(0.044,0.243) | 0.81(0.31,2.15) |  | 0.84(0.34,2.12) |  |
|  | Major | 46 | 0.348(0.214,0.486) | 1.00(0.60,1.67) |  | 0.95(0.58,1.58) |  | 0.087(0.027,0.191) | 0.66(0.23,1.86) |  | 0.61(0.22,1.72) |  |
|  | Bidirectional | 39 | 0.282(0.151,0.429) | 0.85(0.49,1.45) |  | 0.79(0.45,1.38) |  | 0.103(0.032,0.222) | 0.87(0.37,2.08) |  | 0.77(0.31,1.92) |  |
| **CMV** | D-/R- | 47 | 0.468(0.319,0.604) | Reference | 0.22 | Reference | 0.19 | 0.128(0.051,0.240) | Reference | 0.30 | Reference | 0.45 |
|  | D-/R+ | 113 | 0.327(0.243,0.415) | 0.68(0.39,1.18) |  | 0.70(0.41,1.20) |  | 0.142(0.085,0.213) | 1.17(0.47,2.95) |  | 1.19(0.47,3.00) |  |
|  | D+/R- | 27 | 0.407(0.221,0.586) | 0.93(0.46,1.90) |  | 0.99(0.49,2.02) |  | 0.148(0.045,0.308) | 1.20(0.33,4.35) |  | 1.24(0.34,4.56) |  |
|  | D+/R+ | 125 | 0.290(0.213,0.372) | 0.59(0.34,1.03) |  | 0.60(0.35,1.03) |  | 0.081(0.041,0.137) | 0.60(0.22,1.62) |  | 0.65(0.23,1.83) |  |
| **GVHD prophylaxis** | T/S | 205 | 0.348(0.283,0.414) | Reference | 0.44 | Reference | 0.55 | 0.118(0.078,0.166) | Reference | 0.85 | Reference | 0.54 |
|  | PTCy | 107 | 0.327(0.240,0.417) | 0.86(0.58,1.27) |  | 0.89(0.60,1.31) |  | 0.112(0.061,0.180) | 0.92(0.46,1.81) |  | 1.24(0.62,2.49) |  |
| **Year of HCT** | 2016-2019 | 179 | 0.330(0.262,0.399) | Reference | 0.52 | Reference | 0.45 | 0.117(0.075,0.169) | Reference | 0.87 | Reference | 0.99 |
|  | 2020-2021 | 133 | 0.356(0.275,0.438) | 1.11(0.77,1.60) |  | 1.15(0.80,1.65) |  | 0.114(0.067,0.175) | 1.04(0.55,1.96) |  | 1.01(0.53,1.90) |  |
| **MRD** | MRD- | 268 | 0.352(0.295,0.409) | Reference | 0.26 | Reference | 0.30 | 0.120(0.084,0.162) | Reference | 0.55 | Reference | 0.54 |
|  | MRD+ | 44 | 0.273(0.150,0.410) | 0.68(0.38,1.22) |  | 0.74(0.42,1.31) |  | 0.091(0.028,0.199) | 0.70(0.25,1.94) |  | 0.73(0.26,2.02) |  |

* Based on univariate analysis and Gray’s test

† Based on multivariable Fine and Gray regression model adjusted for ELN risk and donor type to Grade II-IV and Grade III-IV aGVHD, respectively.

|  | | | ***Any cGVHD*** | | | | | ***Extensive cGVHD*** | | | | |
| --- | --- | --- | --- | --- | --- | --- | --- | --- | --- | --- | --- | --- |
|  |  | *N* | *1-Yr (95%CI)** | *HR (95%CI)** | *P** | *Adjusted HR (95%CI)†* | *P†* | *1 Yr (95%CI)** | *HR (95%CI)** | *P** | *Adjusted HR (95%CI)†* | *P†* |
| **Age at HSCT, years** | ≤59 | 172 | 0.544(0.466,0.616) | Reference | 0.53 | Reference | 0.75 | 0.521(0.442,0.593) | Reference | 0.23 | Reference | 0.39 |
|  | 60-69 | 102 | 0.475(0.374,0.569) | 0.91(0.67,1.23) |  | 0.92(0.68,1.24) |  | 0.465(0.365,0.560) | 0.94(0.68,1.29) |  | 0.92(0.67,1.27) |  |
|  | ≥70 | 38 | 0.486(0.315,0.638) | 0.77(0.48,1.25) |  | 0.85(0.52,1.40) |  | 0.378(0.223,0.533) | 0.64(0.37,1.08) |  | 0.69(0.40,1.18) |  |
| **Sex** | M | 171 | 0.544(0.466,0.616) | Reference | 0.94 | Reference | 0.48 | 0.503(0.425,0.576) | Reference | 0.92 | Reference | 0.65 |
|  | F | 141 | 0.478(0.392,0.559) | 0.99(0.75,1.31) |  | 0.90(0.68,1.20) |  | 0.464(0.378,0.545) | 1.02(0.76,1.36) |  | 0.93(0.69,1.26) |  |
| **Disease Status** | CR1 | 263 | 0.521(0.459,0.580) | Reference | 0.23 | Reference | 0.43 | 0.498(0.436,0.557) | Reference | 0.077 | Reference | 0.17 |
|  | CR2+ | 49 | 0.478(0.326,0.615) | 0.77(0.49,1.19) |  | 0.84(0.55,1.29) |  | 0.413(0.268,0.552) | 0.66(0.41,1.06) |  | 0.72(0.45,1.15) |  |
| **ELN** | Favorable | 50 | 0.580(0.429,0.705) | Reference | 0.092 | Reference | 0.22 | 0.540(0.390,0.668) | Reference | 0.33 | Reference | 0.54 |
|  | Intermediate | 130 | 0.535(0.445,0.617) | 0.84(0.59,1.18) |  | 0.86(0.61,1.21) |  | 0.504(0.414,0.587) | 0.85(0.58,1.24) |  | 0.85(0.58,1.24) |  |
|  | Adverse | 132 | 0.469(0.380,0.553) | 0.66(0.45,0.95) |  | 0.72(0.50,1.04) |  | 0.445(0.357,0.529) | 0.73(0.50,1.08) |  | 0.80(0.54,1.19) |  |
| **KPS** | 90-100 | 224 | 0.498(0.430,0.562) | Reference | 0.49 | Reference | 0.58 | 0.471(0.403,0.535) | Reference | 0.54 | Reference | 0.51 |
|  | ≤80 | 88 | 0.558(0.445,0.657) | 1.12(0.81,1.54) |  | 1.10(0.79,1.52) |  | 0.523(0.411,0.623) | 1.11(0.79,1.55) |  | 1.12(0.80,1.56) |  |
| **HCTCI** | 0 | 68 | 0.493(0.367,0.607) | Reference | 0.51 | Reference | 0.42 | 0.463(0.339,0.578) | Reference | 0.53 | Reference | 0.54 |
|  | 1-2 | 98 | 0.510(0.406,0.606) | 1.26(0.85,1.86) |  | 1.29(0.88,1.90) |  | 0.490(0.386,0.586) | 1.27(0.84,1.91) |  | 1.26(0.84,1.88) |  |
|  | ≥3 | 146 | 0.528(0.443,0.606) | 1.21(0.84,1.76) |  | 1.21(0.84,1.75) |  | 0.493(0.409,0.572) | 1.19(0.81,1.76) |  | 1.16(0.79,1.70) |  |
| **Conditioning** | MAC | 138 | 0.526(0.438,0.606) | Reference | 0.86 | Reference | 0.55 | 0.496(0.410,0.577) | Reference | 0.91 | Reference | 0.49 |
|  | RIC/NMA | 174 | 0.506(0.428,0.579) | 0.98(0.73,1.30) |  | 0.92(0.69,1.22) |  | 0.476(0.399,0.550) | 0.98(0.73,1.32) |  | 0.90(0.67,1.21) |  |
| **Donor** | Matched | 239 | 0.519(0.453,0.581) | Reference | 0.69 | Reference | 0.067 | 0.498(0.432,0.560) | Reference | 0.22 | Reference | 0.16 |
|  | Not | 73 | 0.500(0.378,0.610) | 0.93(0.66,1.31) |  | 1.48(0.97,2.25) |  | 0.444(0.326,0.556) | 0.80(0.55,1.15) |  | 1.37(0.89,2.11) |  |
| **F to M HCT** | No | 266 | 0.496(0.434,0.555) | Reference | 0.17 | Reference | 0.14 | 0.466(0.404,0.525) | Reference | 0.18 | Reference | 0.13 |
|  | Yes | 46 | 0.622(0.460,0.748) | 1.30(0.86,1.96) |  | 1.35(0.90,2.00) |  | 0.600(0.438,0.729) | 1.31(0.86,2.00) |  | 1.38(0.91,2.09) |  |
| **ABO** | Compatible | 186 | 0.525(0.449,0.594) | Reference | 0.87 | Reference | 0.78 | 0.497(0.423,0.567) | Reference | 0.92 | Reference | 0.73 |
|  | Minor | 41 | 0.450(0.289,0.598) | 0.84(0.53,1.33) |  | 0.79(0.49,1.26) |  | 0.425(0.268,0.574) | 0.87(0.55,1.40) |  | 0.79(0.50,1.26) |  |
|  | Major | 46 | 0.556(0.396,0.689) | 1.07(0.71,1.60) |  | 1.00(0.66,1.53) |  | 0.489(0.334,0.627) | 0.94(0.61,1.44) |  | 0.86(0.56,1.33) |  |
|  | Bidirectional | 39 | 0.487(0.320,0.635) | 0.98(0.61,1.58) |  | 1.02(0.64,1.63) |  | 0.487(0.320,0.635) | 1.06(0.65,1.73) |  | 1.03(0.63,1.66) |  |
| **CMV** | D-/R- | 47 | 0.468(0.319,0.604) | Reference | 0.70 | Reference | 0.52 | 0.447(0.300,0.584) | Reference | 0.92 | Reference | 0.81 |
|  | D-/R+ | 113 | 0.540(0.443,0.627) | 1.13(0.73,1.74) |  | 1.04(0.67,1.63) |  | 0.504(0.408,0.593) | 1.08(0.68,1.70) |  | 1.03(0.65,1.63) |  |
|  | D+/R- | 27 | 0.630(0.411,0.786) | 1.30(0.70,2.41) |  | 1.30(0.70,2.42) |  | 0.556(0.344,0.723) | 1.18(0.62,2.24) |  | 1.26(0.67,2.38) |  |
|  | D+/R+ | 125 | 0.483(0.391,0.570) | 0.99(0.64,1.53) |  | 0.89(0.57,1.40) |  | 0.467(0.375,0.553) | 1.00(0.64,1.56) |  | 0.96(0.61,1.52) |  |
| **GVHD prophylaxis** | T/S | 205 | 0.569(0.498,0.635) | Reference | **0.007** | Reference | **0.002** | 0.554(0.483,0.620) | Reference | **<0.001** | Reference | **0.001** |
|  | PTCy | 107 | 0.410(0.314,0.502) | 0.65(0.46,0.90) |  | 0.54(0.36,0.80) |  | 0.352(0.262,0.444) | 0.54(0.38,0.77) |  | 0.55(0.38,0.78) |  |
| **Year of HCT** | 2016-2019 | 179 | 0.500(0.424,0.571) | Reference | 0.18 | Reference | 0.15 | 0.472(0.396,0.543) | Reference | 0.33 | Reference | 0.35 |
|  | 2020-2021 | 133 | 0.534(0.445,0.616) | 1.21(0.91,1.62) |  | 1.24(0.92,1.66) |  | 0.504(0.415,0.586) | 1.16(0.85,1.57) |  | 1.16(0.85,1.56) |  |
| **MRD** | MRD- | 268 | 0.538(0.476,0.596) | Reference | **0.028** | Reference | **0.019** | 0.511(0.449,0.570) | Reference | **0.028** | Reference | **0.034** |
|  | MRD+ | 44 | 0.372(0.227,0.517) | 0.57(0.35,0.93) |  | 0.55(0.33,0.91) |  | 0.326(0.190,0.469) | 0.56(0.34,0.94) |  | 0.58(0.35,0.96) |  |

* Based on univariate analysis and Gray’s test

† Based on multivariable Fine and Gray regression model adjusted for GVHD prophylaxis and MRD status. Donor type was also included to any cGvHD

**Supplemental Figure 1.** Overall survival by MRD in ELN subgroups.

**Supplemental Figure 2.** Cumulative incidence of relapse by MRD in ELN subgroups.

**Supplemental Table 1.** Multivariate Analysis of Transplant Outcomes.
